# Identification of characteristics of patients who choose operative versus non-operative management after ACL injury: a latent class analysis

**DOI:** 10.1186/s40798-025-00928-4

**Published:** 2025-11-05

**Authors:** Elanna K. Arhos, Joanna Kvist

**Affiliations:** 1 Department of Physical Therapy and Human Movement Sciences, Northwestern University, 645 N Michigan Avenue, Chicago, 60611 IL USA; 2 Unit of Physiotherapy, Department of Health, Medicine, and Caring Science, Linköping University, Linköping, SE-581 83 Sweden

**Keywords:** Anterior cruciate ligament reconstruction, Subgroups, Psychological factors, Social factors, NACOX cohort study

## Abstract

**Background:**

After an anterior cruciate ligament (ACL) rupture, characteristics of patients that may benefit from having ACL reconstruction (ACLR) versus undergoing non-surgical treatment are still largely not known. Identifying patient subgroups may help clinicians and researchers better understand the unique needs of individuals within common clinical profiles. The primary aim of this study was to identify subgroups of patients with an ACL injury, based on patient characteristics, psychological and social factors, and the extent of the initial injury, and to evaluate subgroup characteristics based on undergoing ACLR versus non-surgical management. The secondary aim was to compare subgroups on their knee function and return to sports outcomes.

**Results:**

A total of 275 participants (age 15–40, 48% male) with an acute ACL injury treated with usual care, either ACLR or not, from the Natural Corollaries and Recovery after ACL injury (NACOX) multicenter longitudinal cohort study were included. At two years after injury, 169 (62%) had undergone ACLR. The analysis uncovered 3 latent classes (entropy = 0.91, Akaike’s information criterion (AIC) = 12125.2, Bayesian information criteria (BIC) = 12302.4, sample size adjusted BIC = 12147.0, Vuong-Lo-Mendell-Rubin (VLMR) likelihood ratio test=-6085.8, VLMR p-value = 0.5). Participants in Class 2 (*n* = 155) had 1.8 times (hazards ratio (HR):1.814, 95% confidence interval (CI) 1.293–2.545, *p* < 0.001) higher probability for having an ACLR compared to Class 3 (*n* = 98). There were no statistically significant differences in patient reported knee function (International Knee Documentation Committee (IKDC) score) at one- or two-years follow-up between the classes. A higher proportion of participants in Class 3 returned to same or higher activity level compared to Class 1 (*n* = 22) and 2.

**Conclusions:**

The three distinct subgroups of patients after ACL rupture differed in probability to undergo ACLR and return to activity after injury at one and two years after injury. There was no difference between subgroups in patient-reported knee function at one or two years after injury. Clinicians should consider the differentiating characteristics between subgroups along with the goals of their patients when engaging in shared decision-making about surgical or nonsurgical management.

## Key Points

1. Three patient subgroups were uncovered with significant differences in the decision to undergo ACL reconstruction or non-operative management, as well as in their return to preinjury activity level by two years.
2. Individuals in Class 2, who were the youngest and had the highest pre-injury activity level, self-reported knee function, and motivation to return to sport had a nearly 2 times higher probability for selecting to undergo ACL reconstruction.
3. Individuals in Class 1 made up a small percentage of the subgroups, were predominately male, injured during contact sports, and had the lowest motivation to return to sport and knee self-efficacy, whereas individuals in Class 3 were the oldest, had the highest percentage of females, lowest percentage of contact injuries, primarily participated in IKDC level III sports preinjury and had the highest motivation to return to preinjury activity levels and the highest proportion of participants who did return to the same or higher activity level.

## Background

Anterior cruciate ligament (ACL) injury is a serious knee injury often occurring during sports participation. For athletes participating in contact and pivoting sports, ACL reconstruction (ACLR) is often recommended. While many patients report normal or near normal knee function after ACLR [1], an unacceptably high number of patients experience a challenging recovery after ACLR and the re-injury rates are high [2]. A main reason patients choose to undergo ACLR is to enable successful return to sport, but literature shows only 55–83% of participants return to preinjury sport, with a large variation depending on the sport and level of participation [1, 3]. Non-surgical treatment is a good option for some patients, especially if they do not participate in contact sports [4, 5]. The return to sports rate [6, 7] and patient reported knee function [4, 8] do not differ between the two treatment strategies. Aside from high-level participation in contact and cutting sports, it is unknown what characteristics of patients identify those that may benefit from having ACLR versus undergoing non-surgical treatment.

The decision to undergo ACLR versus non-surgical treatment can be challenging for patients and clinicians alike, and being able to identify characteristics of individuals who may be successful with non-surgical treatment may help guide treatment planning. Long-term knee joint health is also a critical factor to consider in addition to short-term return to activity when planning treatment. Evidence suggests there is no difference in the consequent development of post-traumatic osteoarthritis (PTOA) between those who have underwent ACLR versus those who had non-surgical treatment [8, 9]. Choosing to forgo an ACLR may be advantageous as it avoids surgery, general anesthesia, and additional insult to the knee joint capsule. Regardless, decisions are ideally made using a shared decision-making model where the patient decides their treatment direction with appropriate evidence-based consultation with their rehabilitation specialist and surgeon [10]. 

Identifying subgroups of patients with similar characteristics may help clinicians to recommend treatment strategies that may be more effective for certain individuals after ACL injury. Specifically, understanding those who may be more likely to undergo an ACLR compared to those who are likely to proceed with non-surgical management may help assist clinicians in providing evidence-informed treatment consultation. Subgrouping has been previously used to further understand this clinical population, historically with the classification of copers and non-copers after ACL injury [11, 12]. This refers to patients who can successfully return to activity without having an ACLR by dynamically stabilizing their knee joint through neuromuscular training. Similarly, more recently subgroups have been identified using group-based trajectory modeling based on patient-reported outcome measures (e.g., International Knee Documentation Committee Subjective Knee Form (IKDC-SKF) to identify characteristics of patients with favorable outcomes [13]. 

Latent class analysis is a statistical technique that uses a person-centered approach, which shifts focus away from variable centered analysis where cutoffs are typically used to create subgroups. Using a person-centered approach through latent class analysis captures the complex, multifactorial characteristics that factor into the outcome, which are not fully captured through traditional predictive modeling. Through latent class analysis, unobservable, “latent” constructs are found using observable variables, and homogenous subgroups are uncovered within a heterogeneous sample without an a priori group classification [14, 15]. Recently, latent class analysis has been applied to uncover pre-surgical subgroups and provide initial insight into characteristics that identify individuals at risk for the development of PTOA [16]. These data uncovered four distinct subgroups after ACL rupture that primarily differed by their age and scores on patient-reported outcome measures at baseline [16]. Collectively, these prior studies suggest there are unique subgroups within the larger ACL-injured population that may provide more specific guidance and support towards patient-centered treatment. Thus, the aim of the present study was to identify subgroups of patients with an ACL injury, based on patient characteristics, psychological and social factors, and the extent of the initial injury, and to evaluate subgroup characteristics based on their decision to undergo ACLR versus non-surgical management. The secondary aim was to compare subgroups on their knee function and return to sport outcomes.

## Methods

This is an exploratory analysis of the Natural Corollaries and Recovery After ACL-injury (NACOX) cohort study (trial registration number: NCT02931084), a multicenter longitudinal prognostic cohort study designed to investigate the corollaries and recovery after ACL injury managed with usual care [17]. The study was approved by the Swedish Ethical Review Authority (Dnr 2016/44-31 and 2017/221–32).

Between May 2016 and October 2018, patients who presented to 1 of 7 health care clinics across Sweden (including both public and private clinics) who sustained an ACL injury no more than 6 weeks previously and were between 15 and 40 years old at the time of injury were invited to participate. ACL ruptures were diagnosed by an orthopaedic surgeon and confirmed by magnetic resonance imaging (MRI) or knee arthroscopy. Exclusion criteria included any previous ACL injury to the same knee, serious concomitant injury that required specific treatment (e.g., fracture), inability to understand written or spoken Swedish, cognitive impairments, or other illnesses or injuries that impaired function. Patients provided written informed consent prior to study enrollment.

Patients followed standard of care treatment, which included initial post-injury rehabilitation for approximately 3 months followed by a shared decision between the orthopaedic surgeon and patient on the need for further treatment (i.e., ACLR or non-surgical management), as described by Grevnerts et al. [10] In some cases, there was a decision for early ACLR (within 3 months from the injury) due to larger meniscal injuries (e.g. bucket handle) that had to be repaired or athletes competing at elite level.

Questionnaires, including patient-reported knee-function, symptoms, activity participation, psychological readiness, quality of life, and new injuries/ surgeries, were sent to the participants via a short mobile phone message or email, initially weekly and later monthly to track recovery up to 3 years after injury or ACLR. For this analysis, we analyzed the baseline, 1-, and 2-year follow up timepoints. Baseline refers to the period where patients were enrolled within 6-weeks after injury and had not yet had surgery. Medical charts and radiology reports were reviewed to obtain more information about any new injury or surgery.

### Sample characteristics

For the present exploratory analyses, the following baseline variables were included for the identification of subgroups in the latent class analysis:

Demographics: Age and sex were registered from the specific ten-digit personal identity number. Participants reported weight and height for calculation of body mass index (BMI), previous ACL injury to the contralateral leg (cross-checked in the medical records), and level of sport participation. Sports participation was categorized according to the International Knee Documentation Committee (IKDC) activity levels where level I is pivoting and contact sport, level II is pivoting and non-contact sport, and level III is neither pivoting nor contact sport [18]. Participants also reported which sport their injury occurred during, which was later dichotomized into a contact (e.g., soccer) or non-contact sport (e.g., skiing).

Knee rating: Participants made an overall rating of their knee using the Single Assessment Numeric Evaluation (SANE) [19], answering the question “If I had to give my knee a grade from 1 to 100, with 100 being the best, I would give my knee a ___.’’.

Psychological and social factors: Several patient-reported questionnaires were used. The Swedish version of the General Self-Efficacy Scale (GSES) that assesses the individual’s beliefs that his/her actions determine a successful outcome was used [20]. Motivation for to return to sports was assessed with a single question “How important is it for you to return to your previous sport?”, graded between 1 = not at all important and 10 = very important (MOTIV Importance) [21]. Two out of four questions of the Knee Self Efficacy Scale (KSES) that evaluate patients’ perception of future knee function (“How certain are you that you can participate on the same activity level as before the injury” and “How certain are you that you will not have new knee injuries”, graded 0 = not at all certain and 10 = very certain) were included and the mean score of the answers to these two questions was calculated [22]. The dimensions “lifestyle” and “social and emotional” of the ACL Quality of Life (ACL-QoL) questionnaire [23, 24] were also used. The ACL-QoL consists of 31 total questions across 5 subscales, scored from 0 to 100 where 100 is the best outcome. The “lifestyle” dimension consists of questions regarding general lifestyle outside of work and sports/recreation (e.g., enjoyment of life) and the “social and emotional” dimension consists of questions about attitudes and feelings as they relate to the ACL deficient knee (e.g., apprehensiveness).

### Outcomes

The primary distal outcome in the comparison of the latent classes was undergoing ACLR or not during the first two years after ACL injury. These data were both participant-reported and cross-checked in the medical records and the National Knee Ligament Registry. Secondary outcomes were perceived knee function as reported by the International Knee Documentation Committee Subjective Knee Form (IKDC-SKF) and rate of return to sports. The IKDC-SKF is an 18-item knee-specific questionnaire, covering symptoms, function, and activity level [25, 26]. Return to sports was defined as participating at the same or higher IKDC activity level as before the ACL injury, any time within 1 or 2 years follow up.

### Statistical analysis

Mixture modeling [14, 27] (Mplus) was used to identify the number of latent subgroups present within our cohort. Fit criteria including Akaike’s information criterion (AIC) [28], Bayesian information criteria (BIC) [29], and sample-size adjusted BIC [29] were used to inform selection of the number of subgroups. Having a lower AIC, BIC, and sample-size adjusted BIC signified a stronger model. These fit criteria were supplemented with evaluating class homogeneity (i.e., entropy and tests of model comparison [30, 31]) and clinical relevance and class sizes. An entropy closer to 1 (ranges between 0 and 1) suggests a high level of cohesion within classes and distinction between classes [32]. We applied the criteria that each class should contain at least 5% of the cohort in each group [33]. Finally, Vuong-Lo-Mendell-Rubin (VLMR), Lo-Mendell-Rubin likelihood ratio, and the bootstrap likelihood ratio tests were used to determine whether a model with *k* classes was a stronger model fit than a model with one less class (*k*-1) [31].

Mixture modeling can include both continuous and categorical variables as inputs. The model for this analysis included the 12 previously described baseline variables of age, sex, BMI, SANE, GSES, MOTIV Importance, KSES Mean, ACL-QoL “lifestyle”, ACL-QoL “social”, previous contralateral ACL rupture, participation in contact sport at injury, and IKDC level. Mplus uses a maximum-likelihood estimator to handle missing data during subgroup formation. Assignment to a latent class occurred using each individual’s highest posterior probability.

Once the latent classes were derived, a Cox proportional hazards regression model was fitted on the primary outcome measure of time from injury to ACLR with the latent classes as covariate. The proportional hazards assumption was checked visually in a log-minus-log plot with the latent classes entered as stratum and tested by calculation of the Schoenfeld residuals. The assumptions were met. R package “survival” 3.7-0 was used to fit the Cox proportional hazards regression model and R package “ggsurvfit” 1.1.0 was used to plot the Kaplan-Meier survival curves.

A linear mixed model was used to analyze repeated measures of the secondary outcome of interest (IKDC-SKF score) with time points (baseline, 1- and 2-year follow-up), latent classes and the two-way interaction between time points and latent classes treated as fixed effects. Restricted maximum-likelihood estimate was used in the model, allowing all participants with at least one observation to be included in the model, under the assumption of data missing at random. The unstructured covariance structure was applied in all models to measure the association among the repeated measures. Bonferroni correction was applied on all multiple pairwise contrasts. IBM SPSS Statistics for Windows (version 29.0, Armonk, NY) was used for linear mixed model analysis.

## Results

### Model fit statistics

275 participants were included in the study sample (mean 25.2 years old, 48% male, mean BMI 23.9 kg/m^2^). The analysis uncovered 3 latent classes (entropy = 0.91, AIC = 12125.2, BIC = 12302.4, sample size adjusted BIC = 12147.0, VLMR likelihood ratio test=-6085.8, VLMR p-value = 0.5). Subgroup demographics, reported as probability weighted results, are included within Table 1.

### Latent subgroups

Baseline comparisons between classes are presented in Table 1. Participants in Class 2 were significantly younger and were more active in IKDC-level I sports before the ACL injury than Classes 1 and 3. Participants in Class 1 scored lower on SANE, MOTIV Importance and KSES compared to Classes 2 and 3. There were significantly fewer males in Class 3 and the majority of participants in Class 3 were active in IKDC-level III sports before the injury and had the lowest percentage of injuries occurring during contact sports. Subgroups were not statistically different between having a contralateral ACL injury prior to the current rupture or baseline GSES, ACL-QoL Lifestyle, and ACL-QoL Social scores.

**Table 1:** Participant baseline demographics compared between subgroups. Values are mean ± SD or n (%)

	Total sample (*n* = 275)	Class 1 (*n* = 22)	Class 2 (*n* = 155)	Class 3 (*n* = 98)	*p*-value
Age	25.2 ± 7.0	26.3 ± 5.5	22.5 ± 5.6	29.2 ± 7.5	< 0.001^a, b^
Sex, n (% male)	132 (48)	15 (68)	83 (54)	34 (35)	0.002^b, c^
BMI	23.9 ± 3.2	23.9 ± 1.8	23.5 ± 2.7	24.6 ± 4.0	0.04^c^
Contralateral ACL prior n, (% yes)	22 (9)	4 (18)	12 (8)	6 (7)	0.219
Mechanism of injury, n (% contact sport)	178 (65)	18 (82)	148 (96)	12 (12)	< 0.001^a, b,c^
Concomitant injuries^d^, n (%)	189 (74)	12 (71)	111 (76)	66 (73)	0.779
Meniscus injury	164 (65)	11 (65)	99 (68)	54 (59)	
Other ligament rupture	44 (16)	1 (6)	26 (18)	17 (19)	
Cartilage injury	34 (13)	2 (12)	17 (12)	15 (17)	
New injuries^e^	29 (11)	1 (5)	21 (14)	7 (7)	
Preinjury IKDC Level, n(%)					< 0.001^a, b,c^
IKDC I	158 (58)	16 (73)	142 (92)^a^	0 (0)	
IKDC II	34 (12)	2 (9)	7 (5)	25 (26)^c^	
IKDC III	83 (30)	4 (18) ^a^	6 (4)	73 (75)^b, c^	
SANE	39.1 ± 20.5	30.2 ± 17.4	42.8 ± 21.5	35.3 ± 18.2	0.002^a, b^
GSES	32.4 ± 4.3	33.3 ± 3.8	32.1 ± 4.6	32.6 ± 4.0	0.448
MOTIV importance	9.3 ± 1.5	5.3 ± 1.5	9.5 ± 0.8	9.7 ± 0.7	< 0.001^a, c^
KSES	6.9 ± 1.6	5.6 ± 2.0	7.1 ± 1.6	6.9 ± 1.4	< 0.001^a, c^
ACL QoL- lifestyle	3.9 ± 1.6	3.7 ± 1.6	3.9 ± 1.6	3.8 ± 1.7	0.793
ACL QoL- social	4.5 ± 1.7	4.2 ± 1.8	4.5 ± 1.8	4.5 ± 1.6	0.776

*BMI* body mass index, *ACL* anterior cruciate ligament, *IKDC* International Knee Documentation Committee, *SANE* single assessment numeric evaluation, *GSES* General Self-Efficacy Scale, *MOTIV Importance* Motivation for return to sport, *KSES* knee self-efficacy scale, *ACL QoL* ACL Quality of Life, *MCL* medial collateral ligament, *LCL* lateral collateral ligament

^a^Group differences occurred between Class 1 and 2, ^b^group differences occurred between Class 2 and 3 ^c^group differences occurred between Class 1 and 3,

^d^Concomitant injuries (medial and/or lateral meniscus, total ligament (MCL and/or LCL) rupture, partial or full cartilage lesion) in the ipsilateral knee joint. Data available for 254 patients

^e^New injury was defined as a traumatic injury during an activity-related exposure that resulted in increase of knee symptoms or new symptoms from the knee

### Long-term outcomes

**Fig. 1:**
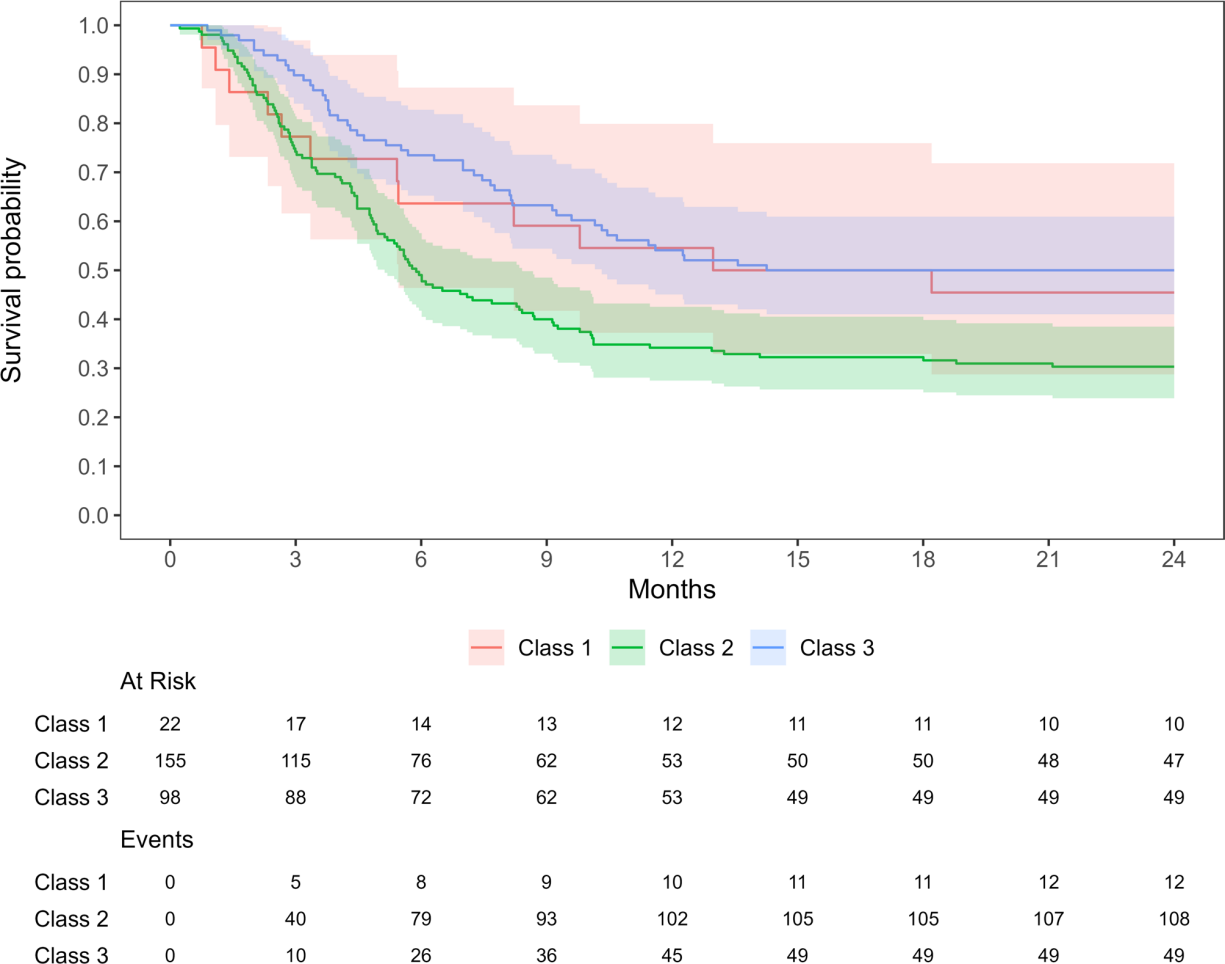
Time from injury to ACLR in the three classes. The table shows the number of patients at risk and events at different time-points. Class 1: average age 26, predominately male, mostly injured in contact sports, primarily IKDC level I preinjury. Class 2: average age 23, about half male, nearly all injured in contact sports and primarily IKDC level I preinjury. Class 3: average age 29, predominately female, most injured during non-contact sports, primarily participated in IDKC level III preinjury

Participants in Class 2 had a nearly 2 times (HR = 1.814, 95%CI 1.293–2.545, *p* < 0.001) higher probability for having an ACLR compared to Class 3 (Fig. 1; Table 2). There were no statistically significant differences in patient reported knee function (IKDC) at one- or two-years follow-up between the classes (Table 2). A higher proportion of participants in class 3 returned to same or higher activity level compared to Class 1 and 2 (Table 3).

**Table 2:** Between-class difference in receiving a treatment (ACLR) by 2-years and patient-reported knee function at 1 and 2 years

	Total sample	Class 1	Class 2	Class 3	Model *p*
**ACLR, n (%)**	169 (62)	12 (55)	108 (70)	49 (50)	0.006^a^
**Time from injury to ACLR (mo)[mean ±SD]**	5.5 (3.9)	6 (5.4)	5.1 (3.8)	6.3 (3.6)	0.204
**IKDC-SKF, mean (95% CI)**					
**1 year**	71 (68–75)	71 (63–80)	75 (71–78)	69 (65–73)	0.130
**2 years**	77 (73–81)	81 (70–92)	77 (73–82)	73 (68–78)	0.338

ACLR, anterior cruciate ligament reconstruction; IKDC-SKF, International Knee Documentation Committee Subjective Knee Form

^a^Group differences occurred between class 2 and 3

**Table 3:** Between-class difference in return to sports

	Total		Class 1		Class 2		Class 3		*p*-value
	n	RTS, n(%)	n	RTS, n(%)	n	RTS, n(%)	n	RTS, n(%)	
1 year									
Total	254	168 (67)	18	8 (45)	140	76 (54)	96	84 (88)	< 0.001^a, b^
IKDC I	140	68 (49)	12	3 (25)	128	65 (51)	0	0	
IKDC II	32	19 (60)	2	1 (50)	6	5 (83)	24	13 (54)	
IKDC III	82	81 (99)	4	4 (100)	6	6 (100)	72	71 (99)	
2 years									
Total	247	204 (83)	15	8 (53)	136	106 (78)	96	90 (94)	< 0.001^a, b^
IKDC I	133	97 (73)	9	3 (33)	124	94 (76)	0	0	
IKDC II	32	25 (78)	2	1 (50)	6	6 (100)	24	18 (75)	
IKDC III	82	82 (100)	4	4 (100)	6	6 (100)	72	72 (100)	

^a^Group differences occurred between Class 1 and 3, ^b^group differences occurred between Class 2 and 3

## Discussion

In the present study we uncovered three distinct subgroups of individuals based on baseline characteristics. Briefly, Class 1 had an average age of 26, were predominately male, included mostly individuals injured during contact sports, and were primarily IKDC level I athletes pre-injury. Class 2 had an average age of 23, were about half male, nearly all were injured during contact sports, and were primarily IKDC level I preinjury. Individuals in class 3 were the oldest, with the average age of 29, predominately female, most individuals injured during participation in non-contact sports, and they were primarily participating in IKDC level III sports pre-injury. The subgroups had significant differences in their treatment strategy as well as in their return to preinjury activity level by two years after injury. Individuals in Class 2 had a 1.8 times higher probability of undergoing ACLR compared to the other two subgroups. These subgroups continue to provide insight into the differentiating factors that may drive some patients to choose to undergo ACLR versus proceed with nonsurgical management.

Class 1 was the smallest (*n* = 22) with individuals being an average age of 26, and the most predominately male subgroup (68%). Individuals in Class 1 had injuries occur mostly in contact sports and were largely IKDC level I preinjury (73%). They also had the lowest SANE, MOTIV Importance, and KSES scores at baseline compared to the other 2 classes, suggesting they had the lowest perception of their knee function and found it least important to return to preinjury sport. Class 2 was the largest (*n* = 155) and youngest subgroup with a mean age of 23 years old. Individuals in class 2 were over half male (54%), nearly all injured during contact sports (96%), and were primarily IKDC activity level I preinjury (92%). These individuals had the highest SANE and KSES scores at baseline, and second highest MOTIV Importance scores (9.5 vs. 9.7 in class 3), suggesting they had a high perception of knee function and found it important to return to preinjury sport. Finally, class 3 was an average age of 29 and had the smallest percentage of males (35%). Unlike the other two classes, individuals in class 3 had the lowest percentage of injuries occurring during contact sports (12%) and primarily participated in IKDC level III sports preinjury (75%). They had significantly lower SANE scores than Class 2, and a significantly higher KSES and MOTIV Importance score than Class 1, suggesting they had a slightly lower perception of knee function and were highly motivated to return to preinjury activity levels.

Another distinguishing factor between these subgroups was age, which has been previously supported by the literature to be a reason patients may choose to undergo surgical versus nonsurgical management. Age is also a reason orthopedic surgeons and physiotherapists may recommend an ACLR [34]. Patients who choose to undergo surgical reconstruction are typically younger and more likely to participate in level I sport prior to injury [6]. Similarly, Sanders et al. found that for every 10-year increase in age there was a 40% reduction in the likelihood of ACLR [35]. Our results may be reflective of the individuals in Class 3 (~ 29 years old, 50% undergoing ACLR) shifting towards activities that are less demanding on knee stability, and potentially individuals in Class 1 (~ 26 years old; 55% undergoing ACLR) making lifestyle decisions that reduce participation in cutting and pivoting sports as well.

The subgroups did not differ in their perception of knee function measured by IKDC-SKF scores either at 1 year or 2 years after injury, and scores ranged from an average of 69 to 75 at 1 year and 73 to 81 at 2 years. IKDC-SKF scores ranged from 0 to 100 where a higher score suggests higher function and fewer symptoms [36], and our results suggest a reasonable patient-reported state [37]. Given the younger age and high level of preinjury IKDC activity level I athletes, along with the high self-reported knee function and motivation to return to preinjury IKDC level, it is not surprising that individuals in class 2 had a nearly 2 times higher probability for selecting to undergo ACLR compared to class 3, where individuals primarily participated in IKDC level III sports (e.g., running or jogging). A large majority (70%) of individuals in Class 2 underwent ACLR approximately 5 months after injury, compared to 55% (Class 1) and 50% (Class 3) who underwent ACLR approximately 6 months after injury.

The influence of sex in receiving ACLR compared to nonsurgical treatment was characterised by mixed findings. One previous study suggested that females have a good prognosis with nonsurgical treatment [38], while others have found no association between patient sex and receiving an ACLR or having a subsequent ACL reconstruction on either knee [35, 39]. In our study, individuals in Class 3 were largely female (65%) and older (29 years) with the lowest probability of having an ACLR. In the overall cohort, 56% of females had an ACLR while 46% underwent non-operative management. While overall more females had ACLR, these data point towards the importance of considering the combination of variables that define each subgroup and not considering each variable separately.

We also assessed the percentage of individuals returning to sport at both 1 and 2 years after injury. Preinjury, Class 1 had an average of 73% participating in IKDC level I sport and 9% in level II. At 1 year, only about 25% of participants returned to IKDC level I sport, with 33% returning at 2 years. 50% (*n* = 1/2) of participants returned to level II sport at 1 year after injury and remained there at 2 years. Class 2 had 92% participating in IKDC level I sport preinjury and 5% in level II. At 1 year, 51% had returned to level I sport, with that number increasing to 76% at 2 years. 83% (*n* = 5/6) returned to level II sport at 1 year with 100% returning by 2 years. Finally, in Class 3 no one (0%) participated in level I sports preinjury and 25% participated in level II. 54% returned to level II sport at 1 year, and 75% returned to level II sport by 2 years. The numbers for IKDC level III were different between the groups. Sports at IKDC level III include running and strength training that are usually part of the rehabilitation program. Preinjury, the distribution of individuals participating in IKDC level III was 18%, 4% and 75% for Class 1, 2 and 3 respectively. At one year, all but one (in Class 3) reported they had returned to the same level and at two years, all had returned. Thus, considering successful return to same IKDC level, Class 3 had a significantly higher proportion of participants returning to the same or higher activity level compared to Classes 1 and 2 (Table 3). These findings are consistent with previous literature that suggests return to sport rates are fairly comparable when assessing matched cohorts and taking preinjury sport level into consideration [40, 41]. While it is frequently assumed that if patients are participating in level I sport they should undergo surgical reconstruction, some patients who are willing to modify their type of activity or participate in less pivoting focused sports favor nonsurgical management.

Interestingly, the subgroups identified in the present study are similar to those identified by Arhos et al. [16], who found four distinct subgroups with similar ages compared to the current study (23, 25, 31, 36 years old respectively). The youngest subgroup had high self-reported knee function at baseline and were primarily level I athletes, while the second subgroup (25 years old) had lower self-reported knee function and a majority were level I athletes 73%. [16] The oldest subgroup (36 years old) was the closest in relation to Class 3 in the current study, with no level I athletes and good self-reported knee function. [16] Clinicians may be able to identify their patients who are represented similarly to those in Class 2 and Class 3 of the current study - the young, competitive athlete eager to return to sport, and the slightly older, recreationally active individual who wants to maintain physical activity level. When considering the individuals in Class 1, the smallest, but still clinically relevant subgroup, it may be more challenging to identify and treat individuals consistent with the characteristics of the subgroup. This group had the least number of individuals returning to their preinjury level I status (33%), but also had the lowest motivation to return to preinjury sport as measured by the MOTIV Importance pre-surgical outcome measure. This may represent a subgroup of individuals who at 26 years old may be transitioning from being a predominately competitive sport athlete to a more recreational level III activity level. When discussing management strategies with patients, it is important to include conversations around the possibility of adjusting activity levels while maintaining quality of life satisfaction, particularly as motivation may change during the rehabilitation period [21, 42]. This Class 1 subgroup may be the best group to suggest non-operative management to along with a dialogue on the willingness to modify activity levels to avoid reinjury.

There are some limitations to consider with the results of our study. First, height and weight were entirely patient-reported. However, while we recognize the potential limitations of self-reported anthropometric data, prior research has demonstrated good agreement between measured and self-reported height and weight [43]. Meniscus injury at the time of ACL rupture is an important factor to consider with surgical decision making, and our latent class model did not include any data on concomitant knee injuries. There were, however, no differences in numbers of concomitant injuries between the three classes. Similarly, we did not include data related to sustaining a new knee injury after the ACL rupture in the latent class analysis. Patients in Class 2 sustained 2 times more new injuries during the follow-up period, compared to Class 1 and Class 3, but the total number of new injuries was too low for statistical comparisons. Further research is needed to understand the chances of sustaining further knee injury both after ACLR and after nonsurgical management with rehabilitation. We also did not have complete data on objective measures of knee function (e.g., strength or hop tests) after injury [44], which has been shown to relate to success with nonsurgical management [38]. We used only two out of four questions of the KSES as the other two questions were not appropriate for our population. The KSES has not been validated for the acute stage after ACL injury. We defined contact versus non-contact injuries by the type of sport participation (classified using IKDC) at the time of injury; therefore, we do not know the exact mechanism by which the injury occurred. Return to sport, defined by the IKDC level of sport participation (i.e., level I and II), does not fully account for the intensity and competition level of the sport participants returned to. Finally, these data did not differentiate between patients who chose to undergo ACLR straightaway versus those who made a delayed decision after nonsurgical treatment failed for them, which may provide a whole separate cohort, perhaps similar to our Class 1 individuals.

## Conclusion

There were three distinct subgroups of patients after ACL rupture that differed in probability to undergo ACLR and return to activity after injury at 1 and 2 year after injury. There was no difference between subgroups in patient-reported knee function at 1 or 2 years after injury. Participants in Class 2 were significantly younger and more active in IKDC-I sports before ACL injury, and also had a higher probability for having an ACLR. Participants in Class 1 had significantly lower motivation to return to sport and patient-reported knee function at baseline compared to Class 2 and 3. Participants in Class 3 were mostly female, participated in IKDC-level III activities before injury and had the highest proportion of participants returning to same or higher activity level compared to Class 1 and 2. Clinicians should consider the subgroup that best represents their patients at baseline to aid in shared decision-making regarding treatment.

## Data Availability

The datasets used and/or analyzed during the current study are available from the corresponding author upon reasonable request.

## Abbreviations

ACL: Anterior cruciate ligament
ACLR: Anterior cruciate ligament reconstruction
PTOA: Post-traumatic osteoarthritis
BMI: Body mass index
IKDC: International knee documentation committee
IKDC-SKF: International knee documentation committee subjective knee form
MRI: Magnetic resonance imaging
SANE: Single assessment numeric evaluation
GSES: General self-efficacy scale
AIC: Akaike’s information criterion
BIC: Bayesian information criteria
VLMR: Vuong-Lo-Mendell-Rubin
MOTIV Importance: Motivation for to return to sports
KSES: Knee Self Efficacy Scale
ACL-QoL: ACL quality of life
